# IDH1 R132H mutation regulates glioma chemosensitivity through Nrf2 pathway

**DOI:** 10.18632/oncotarget.15868

**Published:** 2017-03-03

**Authors:** Kaishu Li, Leping Ouyang, Mingliang He, Ming Luo, Wangqing Cai, Yalin Tu, Rongbiao Pi, Anmin Liu

**Affiliations:** ^1^ Department of Neurosurgery, Sun Yat-sen Memorial Hospital, Sun Yat-sen University, Guangzhou 510120, PR China; ^2^ Guangdong Provincial Key Laboratory of Malignant Tumor Epigenetics and Gene Regulation, Sun Yat-sen Memorial Hospital of Sun Yat-sen University, Guangzhou 510120, PR China; ^3^ Department of Oncology, Sun Yat-sen Memorial Hospital, Sun Yat-sen University, Guangzhou 510120, PR China; ^4^ Department of Pharmacology & Toxicology, School of Pharmaceutical Sciences, Sun Yat-sen University, Guangzhou 510080, PR China; ^5^ Department of Neurosurgery, The Sixth Affiliated Hospital of Guangzhou Medical University, Qingyuan 511500, PR China

**Keywords:** IDH1, Nrf2, NQO1, temozolomide, chemotherapy

## Abstract

**Purpose:**

Numerous studies have reported that glioma patients with isocitrate dehydrogenase 1(IDH1) R132H mutation are sensitive to temozolomide treatment. However, the mechanism of IDH1 mutations on the chemosensitivity of glioma remains unclear. In this study, we investigated the role and the potential mechanism of Nrf2 in IDH1 R132H-mediated drug resistance.

**Methods:**

Wild type IDH1 (R132H-WT) and mutant IDH1 (R132H) plasmids were constructed. Stable U87 cells and U251 cells overexpressing IDH1 were generated. Phenotypic differences between IDH1-WT and IDH1 R132H overexpressing cells were evaluated using MTT, cell colony formation assay, scratch test assay and flow cytometry. Expression of IDH1 and its associated targets, nuclear factor-erythroid 2-related factor 2 (Nrf2), NAD(P)H quinine oxidoreductase 1 (NQO1), multidrug resistant protein 1 (MRP1) and p53 were analyzed.

**Results:**

The IDH1 R132H overexpressing cells were more sensitive to temozolomide than WT and the control, and Nrf2 was significantly decreased in IDH1 R132H overexpressing cells. We found that knocking down Nrf2 could decrease resistance to temozolomide. The nuclear translocation of Nrf2 in IDH1 R132H overexpressing cells was lower than the WT and the control groups after temozolomide treatment. When compared with WT cells, NQO1 expression was reduced in IDH1 R132H cells, especially after temozolomide treatment. P53 was involved in the resistance mechanism of temozolomide mediated by Nrf2 and NQO1.

**Conclusions:**

Nrf2 played an important role in IDH1 R132H-mediated drug resistance. The present study provides new insight for glioma chemotherapy with temozolomide.

## INTRODUCTION

Gliomas are the most malignant of primary brain tumors [1], representing 70% of adult malignant primary brain tumors with a yearly incidence about 0.06% [2]. Gliomas can widely infiltrate normal brain tissue, making it difficult to resect the tumors completely [3, 4]. Current conventional therapies for glioma include surgery, radiation and chemotherapy. Temozolomide (TMZ) is an alkylating agent which can cause methylation on DNA bases in several positions, resulting in DNA lesions and cell apoptosis [5]. Due to its performance in blood brain barrier permeability and its therapeutic effect, TMZ has become the first choice of chemotherapy drugs for glioma treatment [6]. However, resistance to radiation and TMZ chemotherapy is an increasing problem in the treatment of malignant glioma, greatly affecting the prognosis for these patients.

In 2008, mutations in isocitrate dehydrogenase 1 and 2 (IDH1/2) were identified in more than 70% of primary gliomas and secondary glioblastomas (GBM). Patients with the IDH mutation are considered to have a better prognosis than those with IDH wild-type (IDH1-WT) tumors [7–9]. Especially, IDH1 accounted for the largest proportion of IDH mutation cases; more than 90% of the mutations in IDH1 are classed as R132H mutations [10, 11]. Other IDH1 mutations at Arg132 occur at lower frequencies, including R132S and R132L [12]. Functionally, IDH1 catalyzes the conversion of isocitrate to alpha-ketoglutarate (α-KG) and generates nicotine adenine disphosphonucleotide (NADPH) in the cytoplasm and peroxisomes [8]. In addition, IDH1 was involved in a number of cellular functions including glucose sensing, lipogenesis and regulation of cellular redox status [12, 13]. Numerous studies have reported that IDH1 mutation could induce the decline of NADPH and cause reactive oxygen species (ROS) accumulation in cells [14, 15].

Nuclear factor-erythroid 2-related factor 2 (Nrf2) is an important sensor of oxidation in cells; a Kelch-like ECH-associated protein 1 (Keap1) is the negative regulator of Nrf2. Under normal conditions, the Nrf2 protein localizes in the cytoplasm with a low expression level [16]. When cells are under stress, Nrf2 can translocate into the nucleus in active form in order to stimulate the expression of antioxidants, such as phase II detoxification enzymes and other defensive proteins, so as to maintain cell homeostasis [17]. The Nrf2 protein is highly expressed in many cancers, including glioma, to contribute to chemo-resistance. Recent study has also reported that Nrf2 was associated with the prognosis of the glioma patients [18–21]. NAD(P)H quinine oxidoreductase 1 (NQO1) and multidrug resistant protein 1 (MRP1) which are activated by Nrf2 have been proven to be involved in chemical resistance and anti-apoptotic mechanisms [22–24]. More importantly, as an anti-oxidant factor and p53 protector, NQO1 are implicated in the protection of cells against oxidative stress and DNA damage [25, 26]. NQO1 is overexpressed in many cancers. Thus, a high level of NQO1 is also associated with poor prognosis [27, 28]. Moreover, NQO1 is a protector of p53 which is involved in various DNA repair mechanisms. MRP1 is a member of the ATP-binding cassette super family, and in association with the efflux of a broad range of anionic compounds, it is the most intensely studied [29]. MRP1 was first identified in lung cancer tissue, but it is also expressed in many other tissues including kidney, lung, intestine and brain [23, 30, 31]. Overexpression of MRP1 in cancer cells is related to chemotherapy resistance and a poor prognosis [32].

Cells can resist variety of DNA lesions by a series of DNA repairing machineries, and most of the mechanisms are associated with p53. As an important tumor suppressor, p53 protects the genome though a variety of DNA damage response (DDR) mechanisms [33]. On the other hand, P53 can protect glioma cells against DNA damage caused by TMZ.

In this study, we investigated the role of Nrf2 in IDH1 R132H-mediated drug resistance. Our results showed that IDH1 R132H could significantly increase chemosensitivity to TMZ in U87 and U251cells. We also demonstrated that Nrf2 was involved in IDH1 R132H-mediated drug resistance. The role of Nrf2 in IDH1 R132H overexpressing cells provides new insight for glioma treatment in the future.

## RESULTS

### IDH1 R132H mutation significantly increases chemosensitivity to TMZ

**Figure 1:**
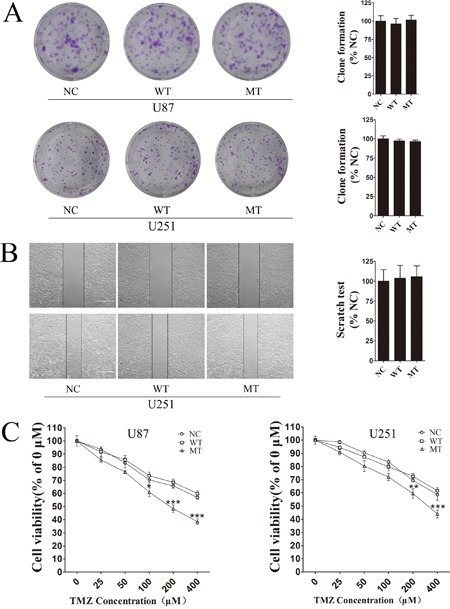
IDH1 R132H mutation increases chemosensitivity to TMZ **(A)** Cell colony formation assay was used to measure cell proliferation in IDH1, WT and NC overexpressing U87 cells and U251 cells. Quantification of cell proliferation is shown on the right. **(B)** Cell mobility difference was assessed using scratch test assay in U251 cells. Quantification of cell mobility is shown on the right. **(C)** IDH1 R132H, WT and NC overexpressing U87 cells and U251 cells were exposed to various concentrations of TMZ for 72 h, and cell viability was measured using MTT assay. All assays were performed in triplicate and representative images are shown. **P < 0.01 and ***P < 0.001 compared with NC and WT.

**Figure 2:**
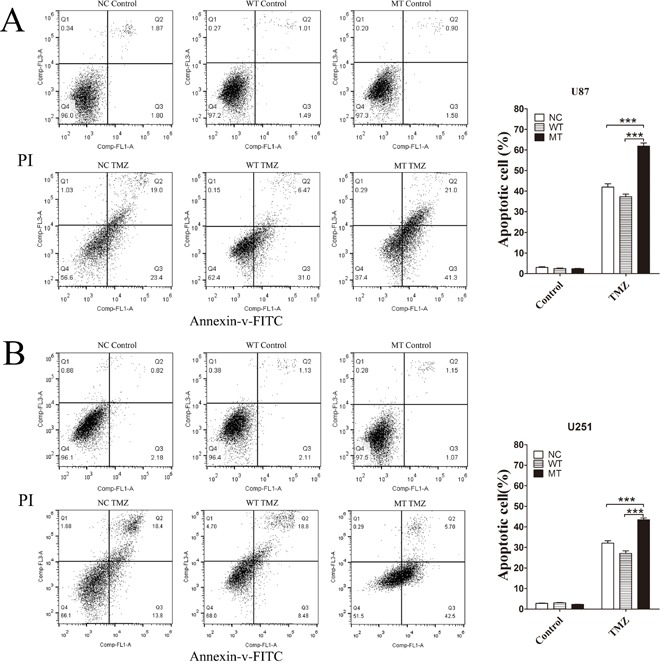
Flow cytometry analysis of cell apoptosis induced by TMZ IDH1 R132H, WT and NC overexpressing U87 cells **(A)** and U251 cells **(B)** were treated with 400 μM TMZ for 72 h and cells without TMZ treatment were served as the control group. Cells were selected with Annexin V-FITC and cell apoptosis was detected by flow cytometer. Assays were performed in triplicate and representative images are shown. Quantification of cell apoptosis is shown on the right. ***P < 0.001 compared with NC, WT.

We evaluated phenotypic differences between NC, IDH1-WT and IDH1 R132H overexpressing U87 cells and U251 cells using cell colony formation assay, scratch test and MTT assay. In cell colony formation assay and the scratch test, no significant differences were observed for cell proliferation (Figure 1A) or cell mobility (Figure 1B) among NC, WT and MT groups. Next, we exposed the NC, IDH1-WT and IDH1 R132H overexpressing cells to various concentrations of TMZ for 72 h for evaluating their chemosensitivity. We were surprised to find that NC and WT overexpressing U87 cells displayed strong chemical resistance to TMZ in the MTT assay; the IC50 was over 400 μM. These results were reproducible in U251 cells. However, IDH1 R132H overexpressing cells were found to be more sensitive to TMZ when compared with NC and WT groups. Respectively, significant differences were observed at 100 μM, 200 μM and 400 μM for U87 cells and at 200 μM and 400 μM for U251 cells (Figure 1C). The difference of chemosensitivity to TMZ reached its maximized point at 400 μM in both U87 cells and U251 cells. This optimal concentration was applied in subsequent experiments. Furthermore, flow cytometry analysis was used to confirm the difference of cell apoptosis between IDH1 R132H and other groups after being treated with 400 μM TMZ. In both U87 (Figure 2A) and U251 cells (Figure 2B), the results of flow cytometry suggested that the apoptosis rates in IDH1 R132H cells were higher than NC and WT overexpressing cells, indicating that IDH1 R132H mutation increases chemosensitivity to TMZ.

### Decreasing Nrf2 expression in cells could increase TMZ sensitivity

**Figure 3:**
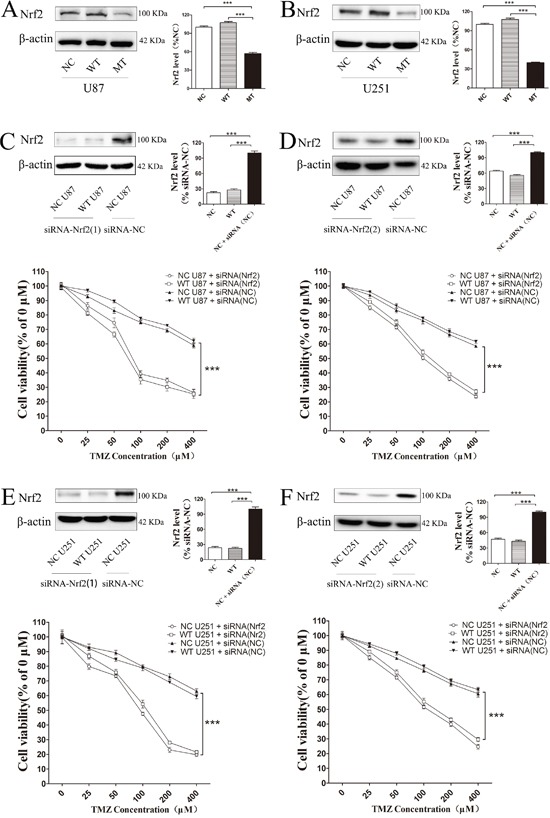
Decreasing Nrf2 expression in IDH1 R132H cells could increase TMZ sensitivity Nrf2 protein level was significantly decreased in IDH1 R132H over expression U87 cells **(A)** and U251 cells **(B)**. Two independent Nrf2-siRNAs were utilized to reduce Nrf2 expression in U87 **(C, D)** and U251 cells **(E, F)**. The chemosensitivity of NC and WT overexpressing cells was significantly enhanced after Nrf2 reduction when cells were exposed to various concentrations of TMZ. ***P < 0.001 compared with NC.

**Figure 4:**
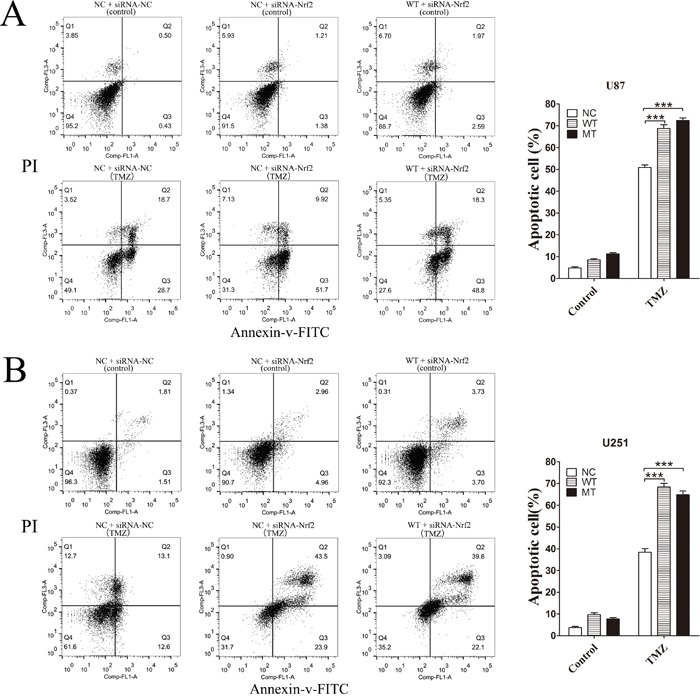
After Nrf2 was knocked down by siRNA-Nrf2 (1), the apoptosis rates of NC and WT cells were tested by flow cytometry again After Nrf2 expression was reduced by siRNA-Nrf2, WT and NC, overexpressing U87 cells **(A)** and U251 cells **(B)** were treated with 400 μM TMZ for 72 h. The cells apoptosis was significantly increased. ***P < 0.001 compared with NC, WT.

Nrf2 protein levels in IDH1 R132H, NC and WT overexpressing cells were measured using Western blotting analysis. Compared with the NC and WT groups, the Nrf2 level was decreased in IDH1 R132H overexpressing U87 cells (Figure 3A) and U251 cells (Figure 3B). In order to confirm the correlation between Nrf2 and the chemo-resistance to TMZ, two independent Nrf2-siRNAs were utilized to reduce Nrf2 expression in U87 (Figure 3C and 3D) and U251 cells (Figure 3E and 3F). The MTT results showed that the chemosensitivity of NC and WT overexpressing cells to TMZ was significantly enhanced after Nrf2 reduction (Figure 3C-3F). Flow cytometry was used to test the apoptosis rates change. After Nrf2 was knocked down by siRNA-Nrf2 (1), the apoptosis rates of NC and WT cells were tested by flow cytometry again. The cells apoptosis was significantly increased after treated with TMZ for 72 h (Figure 4A-4B).

### Decrease nuclear translocation of Nrf2 by IDH1 R132H under TMZ treatment

**Figure 5:**
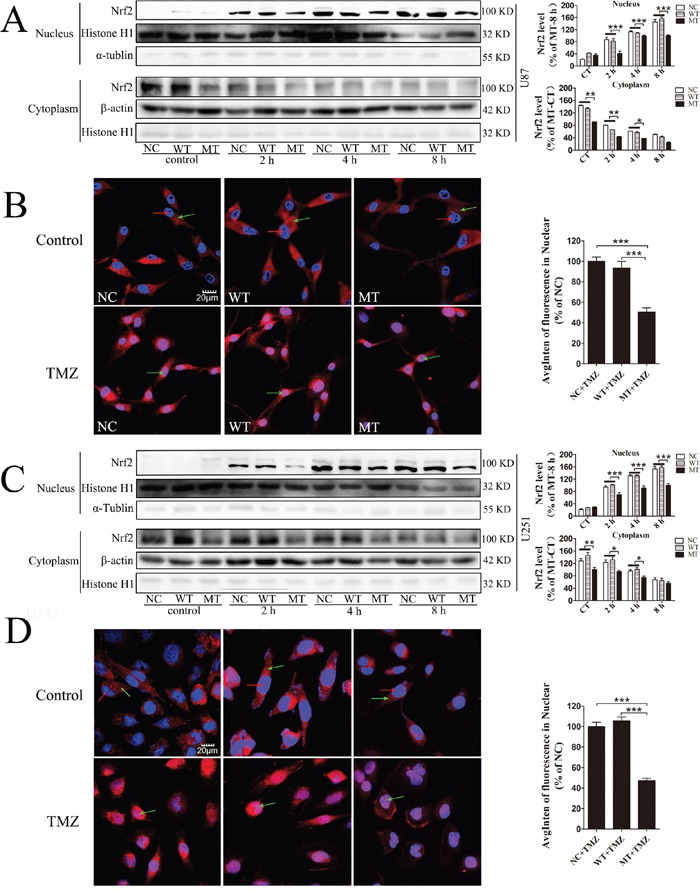
Nuclear translocation of Nrf2 modulated IDH1 R132H under TMZ treatment U87 and U251 cells were treated with TMZ for 0 h, 2 h, 4 h and 8 h. The expression levels of Nrf2 in the nucleus and cytoplasm were measured using Western blotting analysis, and the nuclear translocation level of Nrf2 at different time points was quantified relative to the expression level at 0 h **(A, C)**. In the immunofluorescence assay, Nrf2 protein was stained with red fluorescence and the nuclei was co-stained with DAPI in blue. Green arrows in the pictures indicate the nucleus; red arrows indicate Nrf2 protein expression **(B, D)**. Quantification of Nrf2 in the nucleus is shown on the right. All assays were performed in triplicate and representative images are shown. ***P < 0.001 compared with MT.

Subcellular localization of Nrf2 among NC, WT and IDH1 R132H overexpressing cells was analyzed using Western blotting and immunofluorescence. Cells were treated with 400 μM TMZ for 2 h, 4 h and 8 h, followed by nuclear protein extraction and Western blotting. Nuclear translocation of Nrf2 was increased with the extension of time and Nrf2 level in the cytoplasm was correspondingly reduced. However, in both U87 and U251 cells, nuclear translocation of Nrf2 was lower than that in NC and WT overexpressing cells at the same time point of TMZ treatment (Figure 5A and 5C). After TMZ treatment for 8 h, nuclear translocation of Nrf2 in U87 and U251 cells was observed with confocal microscopy. The Nrf2 protein was stained with red fluorescence and the nucleus was co-stained with DAPI in blue. The results showed that Nrf2 fluorescence intensity in the nucleus was weaker than that in NC and WT in both U87 cells and U251 cells (Figure 5B and 5D), indicating that nuclear translocation of Nrf2 was suppressed by IDH1 R132H.

### ROS level in IDH1 R132H overexpressing cells

**Figure 6:**
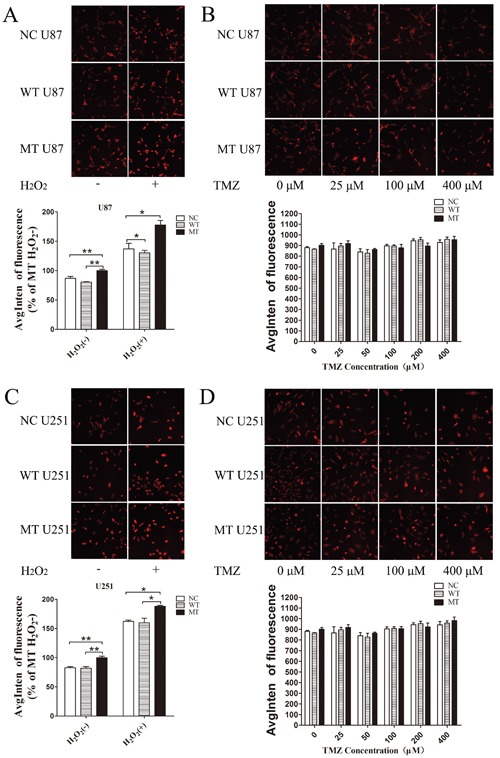
ROS level assayed by high content analysis U87 and U251 cells overexpressing different constructs (NC, WT, IDH1 R132H), treated with hydrogen peroxide, were used as positive control groups; untreated cells were used as negative control groups **(A, C)**. To test intracellular ROS levels induced by TMZ, cells were treated with various concentrations of TMZ for 72 h **(B, D)**; the ROS level was measured using high content analysis. All assays were performed in triplicate and representative images are shown. *P < 0.05, **P < 0.01 and ***P < 0.001 compared with MT.

Compared with NC and WT, the ROS level in IDH1 R132H U87 cells was significantly increased in both the negative and positive control groups, and the results were reproducible in U251 cells (Figure 6A and 6C). However, after treatment with various concentrations of TMZ for 72 h, IDH1 R132H overexpressing U87 and U251 cells did not display a significant accumulation of ROS (Figure 6B and 6D).

### Nrf2, NQO1 and p53 are involved in IDH1 R132H-mediated drug resistance

**Figure 7:**
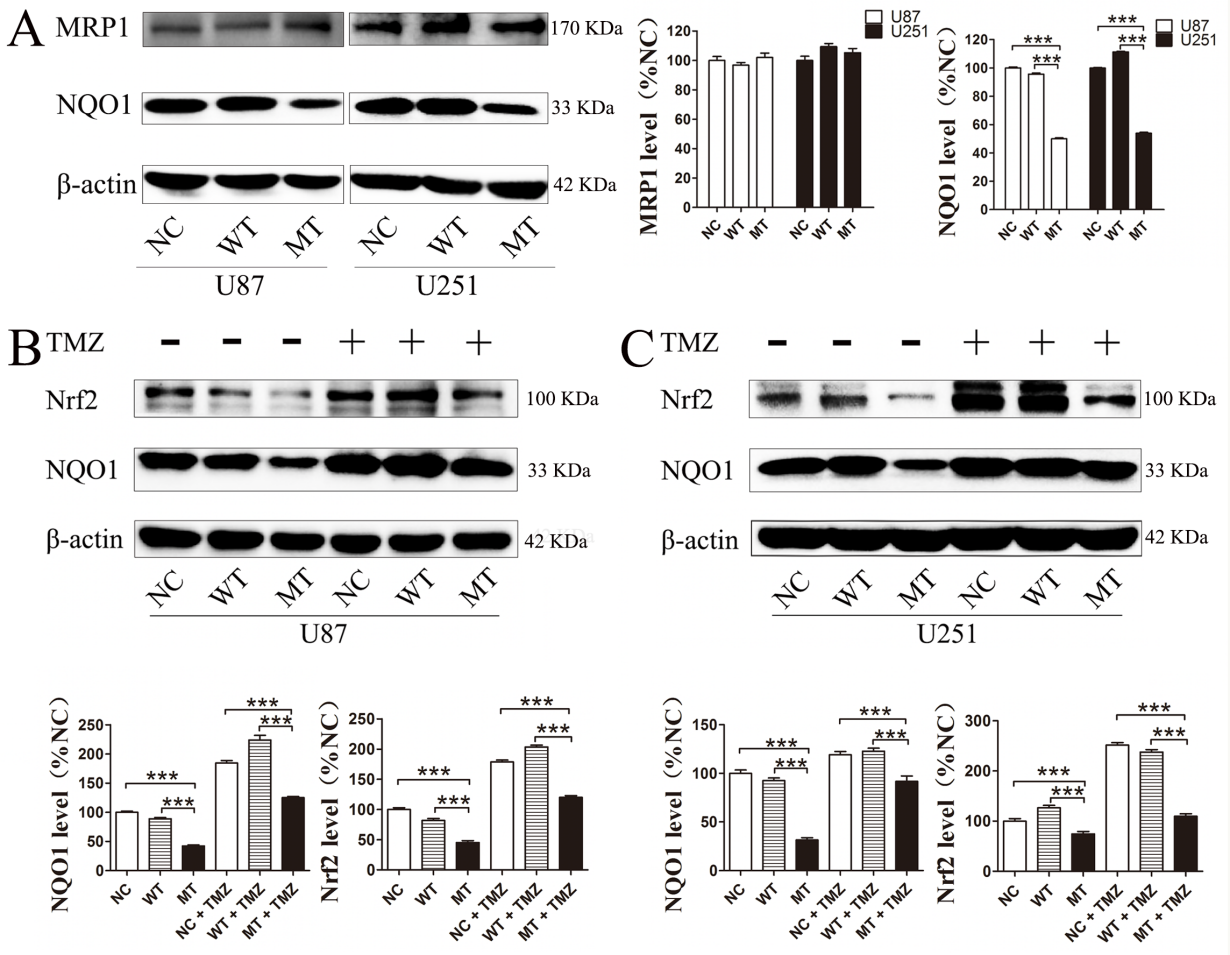
Nrf2, NQO1 and MRP1 proteins level in IDH1 R132H overexpressing U87 cells and U251 cells Base level of NQO1 and MRP1 in U87 and U251 cells **(A)**. After 400 μM TMZ treatment for 72 h, the expression level of NQO1 and Nrf2 in U87 **(B)** and U251 cells was measured **(C)**. Western blotting experiments were performed in triplicate and representative images are shown. Quantification of protein expression is shown at the side or below the images. ***P < 0.001 compared with MT.

**Figure 8:**
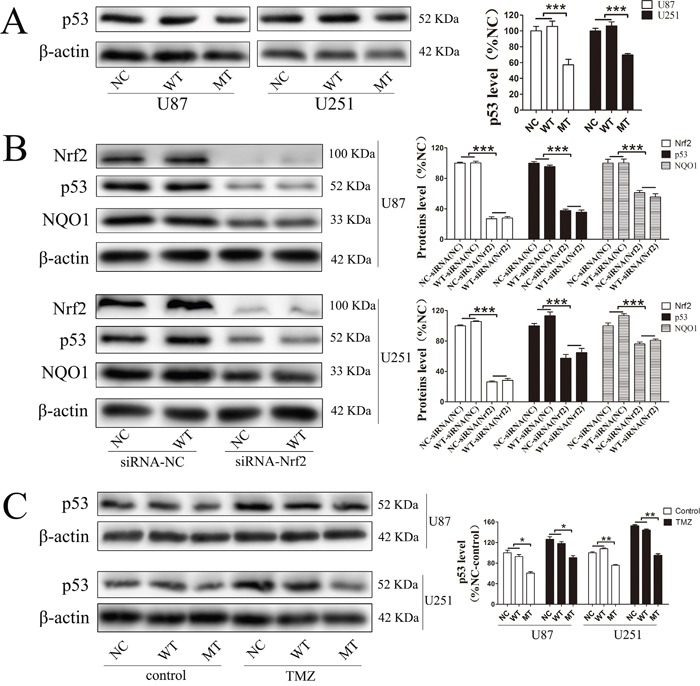
P53 was involved in the resistance mechanism of temozolomide mediated by Nrf2 and NQO1 P53 was significantly decreased in MT groups **(A)**. Levels of NQO1 and P53 were decreased when Nrf2 expression was reduced by siRNA-Nrf2 (1) in NC and WT over-expression cells **(B)**. After treated by 400 μM TMZ for 72 h, p53 was activated in NC, WT and MT groups, and p53 levels in MT groups were still lower than those in other groups **(C)**. *P < 0.05, **P < 0.01 and ***P < 0.001.

In order to confirm whether these downstream proteins are involved in IDH1 R132H-mediated drug resistance, the expression level of these targets in U87 cells and U251 cells was measured using Western blotting analysis. In both U87 and U251 cells, the basal NQO1 level was significantly decreased in IDH1 R132H overexpressing cells while MRP1 was comparable to the NC and WT groups (Figure 7A). We further measured the expression level of NQO1 and Nrf2 in cells after 400 μM TMZ treatment. The results showed that both Nrf2 and NQO1 could be activated by TMZ treatment with a lower level in IDH1 R132H cells than that in NC and WT cells. This indicated that Nrf2 and NQO1 are involved in IDH1 R132H-mediated drug resistance (Figure 7B and 7C). P53 is stabilized by NQO1 and directly involved in various DNA repair pathways. We tested the p35 levels by western blot and the results show that p53 was significantly decreased in MT groups (Figure 8A). Levels of NQO1 and p53 were decreased when Nrf2 expression **was** reduced by siRNA-Nrf2 (1) in NC and WT over-expression cells (Figure 8B). After treated by 400 μM TMZ for 72 h, p53 was activated in NC, WT and MT groups. However, p53 levels in MT groups were still lower than other groups (Figure 8C).

## DISCUSSION

Resistance to radiation and TMZ chemotherapy is the main problem in the treatment of malignant glioma and the prognosis for these patients. Numerous studies have reported that patients with the IDH1 mutation usually receive a better prognosis and have effective TMZ treatment [1, 4, 36, 37]. However, the mechanism of chemotherapy sensitivity in IDH1 mutation cells remained to be understood. In this study, we investigated the role of Nrf2 in IDH1 R132H-mediated drug resistance. We first characterized the proliferation and mobility activity conferred by the IDH1 R132H mutation. Our results showed that IDH1 R132H could significantly increase chemosensitivity to TMZ in U87 and U251 cells instead of affecting cell proliferation and cell mobility. Cell apoptosis rate in IDH1 R132H overexpressing cells was higher than that in WT cells after the treatment with 400 μM TMZ.

IDH1 is involved in energy metabolism. IDH1 dysfunction could induce energy metabolism disorders and cause the accumulation of ROS in cells [38, 39]. Furthermore, many studies also reported that the ROS level in IDH1 R132H cells was increased [15, 40]. Thus, the relationship between the ROS disorder and chemotherapy sensitivity in IDH1 R132H cells was concerned in the past. Nrf2 is also an important antioxidant factor in cells. Nrf2 and its downstream proteins were associated with the chemical resistance of various cancers [22, 41, 42]. Kanamori *et al*. also reported that Nrf2 played an important role in the prognosis of anaplastic glioma patients [42]. In this work, we studied the relationship between ROS, Nrf2 and the chemosensitivity of IDH1 R132H. We found that overexpression of IDH R132H could reduce Nrf2 levels in U87 and U251 cells. This result could be supported by siRNA silencing experiments, showing that the sensitivity of cells to TMZ was significantly increased after Nrf2 silencing, suggesting that Nrf2 played an important role in IDH1 R12H overexpressing glioma cells.

Nrf2 is generally located in the cytoplasm to bind with Keap1 at nonfunctional status. It could translocate into the nucleus and activate downstream proteins under stress conditions. In the present study, both Western blotting analysis and immunofluorescence analysis showed that the translocation of Nrf2 in IDH1 R132H overexpressing cells was significantly lower than that in WT cells, especially after TMZ treatment. In order to test whether ROS was related to the chemosensitivity of TMZ in IDH1 R132H overexpressing cells, we measured the ROS levels. However, both in U87 and U251 cells, ROS did not increase with the increasing concentration of TMZ. Decreasing Nrf2 in IDH1 R132H overexpressing cells did not promote the accumulation of ROS caused by TMZ. Some studies reported that TMZ could induce ROS accumulation in cells and then cause DNA damage (36). However, most studies suggested that the anti-cancer mechanism of TMZ was mainly involved with forced methylation of DNA by the active form of TMZ and MITC (5-(3-methylthree nitrene-1-based) imidazole-4-amide) [43, 44]. Although ROS might be associated with the anti-tumor mechanism of TMZ under certain conditions, ROS is not an important factor involved in Nrf2-mediated chemo-resistance in this study. Therefore, the chemical resistance mechanisms of Nrf2 need to be further identified, such as phase II detoxification enzymes and other defensive proteins which are activated by Nrf2 and related to chemical resistance.

NQO1 and MRP1 are both downstream proteins of Nrf2 and play important roles in cancer chemotherapy resistance [23]. Specifically, NQO1 can protect DNA from alkylation damage [26]. MRP1 is a member of the ATP-binding cassette super family that is associated with the efflux of a broad range of anionic compounds. In this study, the MRP1 level in IDH1 R132H overexpressing cells was comparable to that of the NC and WT groups, although the NQO1 protein level was significantly decreased. We also measured the expression level of NQO1 and Nrf2 in cells after 400 μM TMZ treatment. The results showed that both Nrf2 and NQO1 could be activated by TMZ treatment using lower levels in IDH1 R132H cells than that in NC or WT cells. Overall, these results indicate that Nrf2 and NQO1 are related to IDH1 R132H-mediated drug resistance. However, TMZ can cause DNA damage and induce tumor cells apoptosis. Inoue et al reported that mutant IDH1 could alter DNA repair and sensitivity to DNA damage [45]. Therefore, we further study the DNA protection mechanism of NQO1. P53 is an important protective factor involved in most of DNA protection mechanism. As a genome protector, p53 can utilize a series of mechanisms against DNA damage caused by TMZ. More importantly, p53 can be stabilized by NQO1 [46]. In this study, we found that p53 was significantly decreased in the MT groups and Nrf2 could activate p53 expression through NQO1. Although p53 was activated by TMZ, the level of p53 in MT groups was still lower than other groups.

However, the regulatory mechanism between IDH1 mutation and Nrf2 remained unclear. As an important energy metabolism enzyme, IDH1 mutation could cause a series of disorders of energy metabolism. Thus, we hypothesize that the metabolism change might affect Nrf2 protein expression. Moreover, 23 miRNAs had been reported to be differentially expressed in IDH1 mutant and IDH1 wild-type patients [47], and some of them are involved in the regulation of Nrf2. Above all, regulatory mechanisms between IDH1 and Nrf2 are complicated and need more research to reveal their relationship.

At present, numerous studies are taking the IDH1 mutation as a therapeutic target [1, 48], and several IDH inhibitors have been proved effective *in vitro* and *vivo* [49, 50]. Targeted therapy for IDH1 mutation has displayed great potential in cancer therapy. However, no inhibitors have been reported that they can produce a synergistic effect with temozolomide in the treatment of glioma. Thus, the mechanism of IDH1 mutation sensitivity to chemotherapy should be further researched and people should provide new ideas to promote IDH1 mutation inhibitors exploration and glioma treatment.

## MATERIALS AND METHODS

### Cell lines and cell culture

Human malignant glioblastoma (GBM) cell lines, U87 cells and U251 cells were purchased from the Shanghai Institute of Biochemistry and Cell Biology (Shanghai, China). All these cell lines were cultured and maintained in DMEM (Gibco-BRL, NY, USA) supplemented with 10% fetal bovine serum (FBS) (Gibco-BRL NY, USA) and incubated at 37°C with a 5% CO_2_ humidified atmosphere. Cells were passaged at 80% confluence.

### Plasmid construction and lentivirus transfection

LV5 shuttle plasmid (Genepharma, Suzhou, China) was employed as a lentivirus vector. IDH1 R132H mutation was introduced by site-directed mutagenesis, referring to IDH1 wild type sequence (GenBank ID: NM_005896). IDH1 R132H mutation was occur on the 132nd amino acid of the IDH1 gene, which was mutated to histidine (CGT to CAT). The cDNA was fused in-frame with a FLAG tag at the N-terminus using the following synthesized primers: forwad primer with NotI site 5′-GATAGGCGGCCGCGCCACCATGGATTACAAGGATGACGACGATAAGTCCAAAA-3′, and reverse primer with BamHI site 5′-CTATGGGATCCTTAAAGTTTGGCCTGAGCTAGTTT-3′. The amplified target was inserted into the NotI and BamHI linearized LV5 vector. R132H, forward primer 5′-CATCATAGGTCATCATGCTTATGGGGATCAATACAGAGCAACTGA-3′, revers eprimer 5′-CATAAGCATGATGACCTATGATGATAGGTTTTAC CCATCCACTCAC-3′. LV5 shuttle plasmid without carrying IDH1 gene sequence was used as the negative control (NC) lentivirus vector. U87 cells and U251 cells were cultured in 35mm dishes. The culture medium was replaced with fresh DMEM without FBS when cells were at 80% confluence. Then 10 μL lentivirus solutions were added to the medium and incubated for 24 h. After 3 days of transfection, transfected cells were selected and enriched by applying 2 μM puromycin (Selleck, Shanghai, China) in the culture medium for 24 h (un-transfected cells were all apoptotic after 0.5~1 μM puromycin treatment for 24 h). Then, the adherent cells continued passage. When the cells fusion reached 80%, the cells were treated with elevated concentrations of puromycin again. After 4 weeks, the cells could resist 4 μM puromycin treatment. GFP expression was assessed by fluorescence microscopy to confirm that the stable cell lines have been established. Flag protein expression was measured by employing Western blot analysis to evaluate transfection efficiency.

### Cell colony formation assay

U87 cells and U251 cells were seeded onto a 6-well plate at a density of 50-60 cells per well. After 2 weeks, cells were washed with PBS and subsequently fixed with 5 ml methanol for 20 min and stained with Giemsa staining solution for 30 min. The stained cells were washed with PBS and observed through a low power lens. A colony with more than 50 cells was counted. The results were expressed as a percentage of the NC group (NC lentivirus vector over expression cells), which was set as 100%.

### Scratch test assay

Cell migration ability was assessed using the scratch test assay. U251 cells were seeded onto a 6-well plate at a density of 2 × 10^5^ cells/ml and cultured for 24 h. A linear scratch was made with a 200-μl sterile pipette tip when cell confluence reached 100%. Cells continued to grow in serum-free DMEM for another 24 h, and scratched wound healing was observed by microscope. The recovery distance of the scratch was compared with the NC group (NC lentivirus vector over expression cells).

### MTT assay

Cell viability was determined by using MTT assay. Briefly, cells were cultured on 96-well plates. After being treated with TMZ at different concentrations for 72 h, MTT (5 mg/mL) was added to the cells and the mixture was incubated for 2 h at 37°C. MTT reagent was then replaced with DMSO (100 μL per well) to dissolve formazan crystals. After the shaking mixture at room temperature for 10 mins, absorbance was determined at 570 nm using a microplate reader (Bio-Tek, USA). Cells without TMZ treatment were used as the control group. Results were expressed as a percentage of the absorbance of control cells, which was set as 100%.

### Flow cytometry

Flow cytometry was utilized to evaluate cell apoptosis. Cells were treated with 400 μM TMZ for 72 h. Cells without TMZ treatment were served as the control group. Afterwards, cells were gathered and treated with an Annexin V-FITC apoptosis detection kit (Keygen Biotech). Briefly, cells were washed with PBS three times and re-suspended in a 400 μl binding buffer, then incubated with 5 μl of Annexin V-FITC and 5 μl of PI at room temperature in the dark for 15 min. Finally, cell apoptosis was detected using flow cytometry (Gallios, Beckman-coulter, USA) within 1 h.

### Knock down with siRNA

Two siRNAs of Nrf2 were designed by Genepharma (Suzhou, China) according to the Nrf2 sequence (GenBank ID: NM_006164). The sequence of siRNA1 is sense 5′-AGAUUUAFAUCAUUUGAA-3′, anti-sense 5′-UUUCAAAUGAUCUAAAUCUTG-3′. The sequence of siRNA2 is sense 5′-ACAGUGUCUUAAUAUUGAATT-3′, anti-sense 5′-UUCAAUAUUAAGACACUGUAA-3′. The sequence of siRNA negative control (siRNA-NC) is sense 5′-UUCUCCGAACGUGUCACGUTT-3′, anti-sense 5′-ACGUGACACGUUCGGAGAATT-3′. U87and U251 cells were cultured on 35mm dishes. SiRNAs were transfected into cells using lipofectamine 3000 reagent (Thermo, USA) when cells were at 80% confluence.

### Western blotting analysis

Cells were cultured in 60mm or 35mm dishes. Before protein extraction, cells were washed twice with cold PBS, then suspended in 80 μL of RIPA lysis buffer containing protease inhibitor. Protein concentration was determined using BCA assay (Thermo Fisher Pierce, Rockford, USA). Nuclear extraction was separated using a nuclear and cytoplasmic protein extraction kit (Beyotime, Shanghai, China). 20 μg of nuclear protein was loaded in each lane and resolved using SDS-PAGE gel electrophoresis followed by transfer onto a PVDF membrane (Millipore, Billerica, MA). After blocking with 5% skimmed milk, the membrane was probed with specific antibodies, including anti-histones H1 (1:1000, Abcam, Shanghai, China), Nrf2 (1:500, Santa Cruz, Dallas, USA), NQO1 (1:1000, Santa Cruz, Dallas, USA) and MRP1 (1:1000, Novus Biologicals, NY, USA). β-actin primary antibody (1:10000, Thermo, MA, USA) and α-tublin primary antibody (1:3000, Thermo, MA, USA) were used as the loading control. Horseradish peroxidase-conjugated rabbit and mouse secondary antibodies were reacted with the membranes, respectively. Immunoblot bands were detected by the enhanced chemoluminescence technique. Image J software was utilized to quantify the grey intensity.

### Immunofluorescence

Cells were cultured on coverslips and incubated for the indicated treatments. Cells were fixed with 4% paraformaldehyde for 15 minutes followed by blocking with 10% FBS, with 0.1% Triton X-100 for 30 minutes. Nrf2 primary antibody (1:50) was added onto coverslips overnight at 4°C and then incubated with secondary antibodies at room temperature for 1 hour. Cell nuclei were co-stained with 0.4% DAPI for 15 minutes. The images were captured using a laser-scanning confocal microscope (Zeiss, Germany). Image-Pro Plus software was utilized to analyze the fluorescence intensity.

### ROS analysis

Cells treated with hydrogen peroxide (H_2_O_2_) were used as the positive control; cells without H_2_O_2_ treatment were used as the negative control. In the positive control group, cells were treated with 300 μM H_2_O_2_ for 15 minutes. Cells expressing WT or MT IDH1 were cultured on 96-well plates, then treated with TMZ for 72 h. The medium was then replaced with DMEM, containing 10 μM dihydroethidium (DHE). The samples were detected with high-content screening system [34, 35] (Thermo Fisher) to calculate the fluorescence intensity, which can reflect intracellular ROS levels.

### Statistical analysis

All experiments described in this study were repeated at least three times. The data was presented as the mean ± S.D. Statistical analyses between the two groups performed by unpaired Student’s *t* test. Differences among groups were tested by one-way analysis of variance (ANOVA). Followed by ANOVA analyses, the Tukey test was used. P < 0.05 was considered to be statistically significant.
